# Patient reported outcomes – experiences with implementation in a University Health Care setting

**DOI:** 10.1186/s41687-018-0059-0

**Published:** 2018-08-17

**Authors:** Joshua Biber, Dominik Ose, Jenny Reese, Anna Gardiner, Julio Facelli, Joshua Spuhl, Darrel Brodke, Vivian S. Lee, Rachel Hess, Howard Weeks

**Affiliations:** 1 University of Utah Medical Group, Salt Lake City, UT USA; 2 Department of Population Health Sciences, Division of Cancer Population Science, University of Utah, Salt Lake City, UT USA; 3 Department of Biomedical Informatics, University of Utah, Salt Lake City, UT USA; 4 Center for Clinical and Translational Science, University of Utah, Salt Lake City, UT USA; 5 Enterprise Data Warehouse|, University of Utah, Salt Lake City, USA; 6 Department of Orthopaedics, University of Utah, Salt Lake City, UT USA; 7 Department of Radiology, University of Utah, Salt Lake City, UT USA; 8 Department of Population Health Sciences, Division of Health System Innovation and Research, University of Utah, 295 Chipeta Way, Williams Building Room 1N492, Salt Lake City, Utah UT 84102 USA; 9 Department of Internal Medicine, Division of General Internal Medicine, University of Utah, Salt Lake City, UT USA; 10 Department of Psychiatry, University of Utah, Salt Lake City, UT USA

**Keywords:** Patient reported outcome measures, Information systems, Patient-centered care, Hospital administration

## Abstract

**Aim:**

Patient-reported outcomes (PROs) have traditionally been implemented through a manual process of paper and pencil with little standardization throughout a Healthcare System. Each practice has asked patients specific questions to understand the patient’s health as it pertains to their specialty. These data were rarely shared and there has not been a comparison of patient’s health across different specialty domains. We sought to leverage interoperable electronic systems to provide a standardization of PRO assessments across sites of care.

**Methods:**

University of Utah Health is comprised of four hospitals, 12 community clinics, over 400,000 unique annual patients, and more than 5000 providers. The enterprise wide implementation of PROs started in November of 2015. Patients can complete an assessment at home via email, or within the clinic on a tablet. Each specialty has the opportunity to add additional specialty-specific instruments. We customized the interval with which the patient answers the assessments based on specialty preference in order to minimize patient burden, while maximizing relevant data for clinicians.

**Results:**

Barriers and facilitators were identified in three phases: Pre-implementation, Implementation, and Post-implementation. Each phase was further broken down into technical challenges, content inclusion and exclusion, and organizational strategy. These phases are unique and require collaboration between several groups throughout the organization with support from executive leadership.

**Discussion:**

We are deploying system-wide standard and customized PRO collection with the goals of providing better patient care, improving physician-patient communication, and ultimately improving the value of the care given. Standardized assessment provides any clinician with information to quickly evaluate the overall, physical and mental health of a patient. This information is available real time to aid in patient communication for the clinician.

**Electronic supplementary material:**

The online version of this article (10.1186/s41687-018-0059-0) contains supplementary material, which is available to authorized users.

## Introduction

In recent years, there has been an increased interest in understanding the value and use of Patient Reported Outcomes (PROs) in clinical care [1–4]. PROs are directly reported by the patient, reflect the patient’s health-related quality of life and functional status, and provide insight into the patient’s perspective of their health [5–7]. The use of PROs can empower patients to take an active role in their care [8] and effectively communicate their health status [9, 10]. PRO scores can also be aggregated and used to evaluate the comparative effectiveness of treatment options [11, 12] and inform policy decisions [13, 14].

The adoption of PROs in healthcare systems remains limited [15]. Technical and logistical challenges have limited deployment of PROs broadly within health care systems. At University of Utah Health, we are implementing the collection, reporting, and analytical use of PROs throughout the clinical enterprise. Currently, PROs collection is in place for about 75% of the ambulatory encounters within our system. Facilitators of this program include collaborating with our Information Technology (IT) team to create a home-grown application, integration of data into our Electronic Health Record (EHR), and provider champions.

Engaging patients and managing change in an organization that has four hospitals, 12 community clinics, over 400,000 unique patients on an annual basis, and more than 5000 practicing clinicians is difficult. In this paper, we describe our experiences implementing PROs including significant facilitators and barriers to PRO implementation that may benefit other academic medical centers in similar implementations.

## Methods

Implementation of electronic PRO collection at University of Utah Health began in 2013 at the University Orthopaedic Center. In 2015, we began the health system-wide implementation our custom-designed platform, My Evaluation (mEVAL). We created our own platform to allow for customization and reduce vendor-imposed constraints. mEVAL is built within our Enterprise Data Warehouse with 3.0 total full-time equivalent (FTE) personnel allocated for maintenance and continued implementation. In 2016, we established the goal of reaching all ambulatory care practices by June 2018. We are currently collecting more than 15,000 assessments each month in 75 different practices ranging from primary care to sub-specialty surgical practices.

**Fig. 1:**
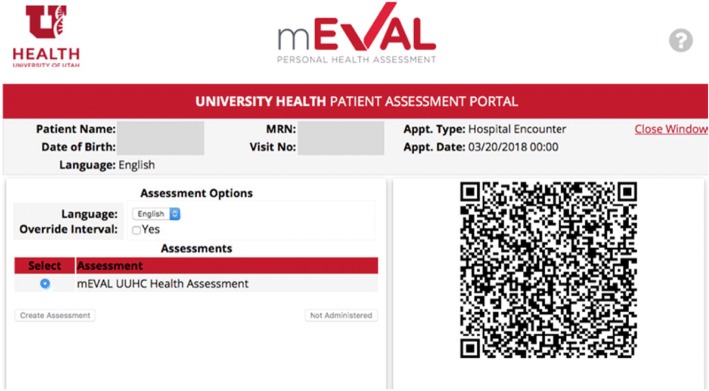
mEVAL Portal Used by Staff to Sign Patients in to the mEVAL assessment

PROs are administered one of two ways: [1] ahead of the visit at home, via secure email link, or [2] in clinic on a tablet computer. In clinic, a staff member uses the mEVAL secure web-portal (Fig. 1), which includes every patient in our healthcare system, to generate an assessment, which is then completed by the patient on a tablet computer. The mEVAL portal is only used by the staff and does not require any login information from the patient. The mEVAL portal identifies patients eligible to complete PROs based on the patient schedule in the EHR and assigns the appropriate PRO instruments to the patient for completion based on the provider and visit department. If needed (e.g., the patient is a walk-in and not scheduled), the staff can also generate an ad hoc assessment for the patient.

**Fig. 2:**
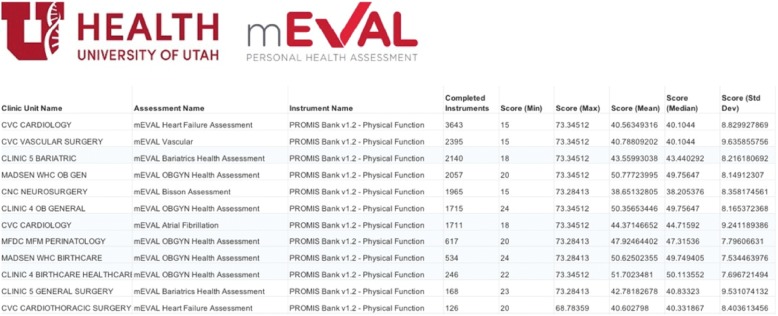
Self-Service Portal Used by Providers to Compare PROs across populations

In the EHR, clinicians are able to view patient-reported PRO information in summary scores, longitudinal graphs and individual questions and answers. These options allow each clinician to access the data they find most important at that time. Providers also have access to aggregated scoring data across their patients and can compare those data to their peers within the health system or against a national average (Fig. 2). We do not currently make the scores directly available to the patient because we feel it is important for the clinician to discuss how the scores are interpreted, but do offer the patient supplemental information (Additional file 1). We are developing and testing strategies to provide PRO scores directly to patients that include clear interpretation information.

Metrics of project success include the percentage of eligible patients who complete PROs at each practice and percentage of practices reaching the threshold of 80% of eligible patients completing PROs.

The study of the implementation of PROs was reviewed by the University of Utah Institutional Review Board, was deemed not to meet the definition of human subjects research, and was exempt from institutional review board oversight.

## Results

We assessed the barriers and facilitators for each of the three phases: pre-implementation, implementation, and post-implementation (Table 1). For each, we consider technical challenges, content, and organizational strategy.

**Table 1:** Barriers and facilitators for each of the three implementation phases: pre-implementation, implementation, and post-implementation

	Pre-Implementation	Implementation	Post-Implementation
Technical considerations	− Determine method of PRO delivery − Evaluate and decide on tablet device − Preparing IT infrastructure − Develop GUI for administration − Integration into EMR	− Add providers to portal − Configure tablets − Test the assessments	− Use release cycles for upgrades − Consider use of foreign languages Make data available in different analytic platforms
Content aspects	− Determine assessment standard (e.g., PROMIS) − Create institution “core” assessment − Consider the use of specific instruments − Create implementation checklist for clinics	− Train staff and clinicians − Create education cards − Follow implementation checklist	− Provide completion metrics − Create self-service portal − Collaborate with research initiatives
Organizational strategy	− Create Executive Steering Committee − Develop project charter − Identify clinic champions − Engage support teams, e.g., marketing − Determine assessment intervals	− Collect relevant metrics − Create executive level reporting dashboards − Executive promotion	− Create marketing for implementation − Provide executive level metrics − Integrate into organizational goals
Staff training	− Create training materials (e.g., education cards) − Develop computer based training for employees − Perform hands-on training with staff − Explain trouble ticket submission process − Inform providers of go-live	− Provide additional training during go-live − Walk staff through workflow during patient encounter − Demonstrate results within EMR	− Follow up with staff at 1 week, 1 month and 3 months − Answer “pop-up” questions and modify training material accordingly
Technical Support	− Create asset management model with established product life cycle for tablets − Create “gold image” for the tablet with the use of a Mobile Device Manager − Set up IT trouble ticket support system	− Delivery of tablets and storage unit with integrated charging − Go-live support for any connectivity issues	− Scheduled application or tablet upgrades / updates − Replacement of malfunctioned devices or cases − Continued support for any unexpected issues

### Phase 1: Pre-implementation

#### Technical considerations

The mEVAL platform provides patients with two ways to complete the PRO assessment. Seven days prior to the appointment, a patient receives a secure email with a unique link to the PRO questionnaire and can complete it at any time up to the time of the appointment, with 72% of our patients having a valid email address. In the clinic, the patient is also able to complete the assessment on a tablet computer. Overall, we are below our goal with 47% of patients complete a mEVAL assessment, 17% completing them at home and 30% completing them in clinic.

When completing the assessment remotely, the unique link in the secure email does not require the patient to input of any information, such as name or birthday. Once in clinic, the staff uses a Quick Response (QR) code to start the patient’s assessment, specific to the clinic or physician being seen. To facilitate provider use of the completed PRO assessments, scores are uploaded into the EHR within 1 minute of completion, allowing the information to be viewed by clinicians during the same clinic visit.

#### Content

We created a system-wide “core assessment” to gain a better understanding of the patient’s overall health-related quality of life and provide the potential for population health assessment. The core assessment was designed by a panel of PRO subject matter experts and is completed by patients at least annually and no more often than weekly. It includes a general health question, a current health visual analog scale and PROMIS-Bank v1.2 Physical Function and PROMIS-Bank v1.0 Depression [16]. Physical Function and Depression were chosen to evaluate overall physical and mental health of the patient [17–19]. Other domains, like Anxiety, were also considered. This core assessment can be supplemented by specialty-specific PROs selected by the clinical department and vetted by medical subject matter experts and through review of relevant literature. PROMIS instruments were chosen for their wide applicability and the brevity and responsiveness afforded by the computer adaptive testing algorithms, which selects the next question based on how the patient responded to the previous question. We identified specific thresholds for common instruments, such as PROMIS Depression, in order to allow for easier scoring interpretation and standardization throughout the healthcare system. In total, more than 200 different instruments are in use, with the core assessment implemented in each clinic.

#### Organizational strategy

Leadership support was crucial in advocating for the use of PROs and facilitating an enterprise adoption process. We identified key stakeholders for an Executive Steering Committee, representing Quality, Strategy, Operations, and Information Technology. This committee helped to develop a project charter, which defined the scope of the project, measures of success, and the project timeline. The Executive Steering Committee also helped identify and engage support teams including Marketing, Asset Management for tablet support, IT Training, and others.

Physician champions were identified for each clinic. These champions advocate for the use of PROs, identify specialty-specific PROs, and assist project teams in identifing and removing implementation barriers. They were the clinical expert leads demonstrating clinical use of the data to help support adoption within the clinic. The physician champions were also instrumental in organizing clinician training and education regarding scoring interpretation and standardized scoring thresholds used throughout the healthcare system.

### Phase 2: Implementation

#### Technical considerations

When implementing PROs within a clinic, it was essential to assign the appropriate providers and clinics to the correct questionnaires. This is particularly important if a provider is associated with more than one clinic, as is the case for many of our oncology providers, for example. This ensures that a patient receives the correct assessment associated with their diagnosis, provider, and practice.

#### Content

When developing a home-grown PRO platform, each instrument must undergo careful pre-release testing to ensure that the content and scoring algorithm are accurate and instruments are displayed as intended.

#### Organizational strategy

Our executive dashboard included metrics around overall completion percentage, number of assessments completed by month, as well as a running total of completed assessments. This dashboard allowed for the overall progress of the project to be viewed on one screen. While the overall completion percentage is less than 80%, some clinics have completion percentages greater than 90%. This disparity has prompted the discussion of promoting provider adoption within clinics with lower completion percentages.

### Phase 3: Post-implementation

#### Technical considerations

We implement upgrades on a quarterly release cycle instead of releasing individual upgrades on an ad hoc basis. This allows us to plan when the different functionalities will be ready, provide training and communication to existing users, and more efficiently use personnel resources.

#### Feedback and PRO use

**Fig. 3:**
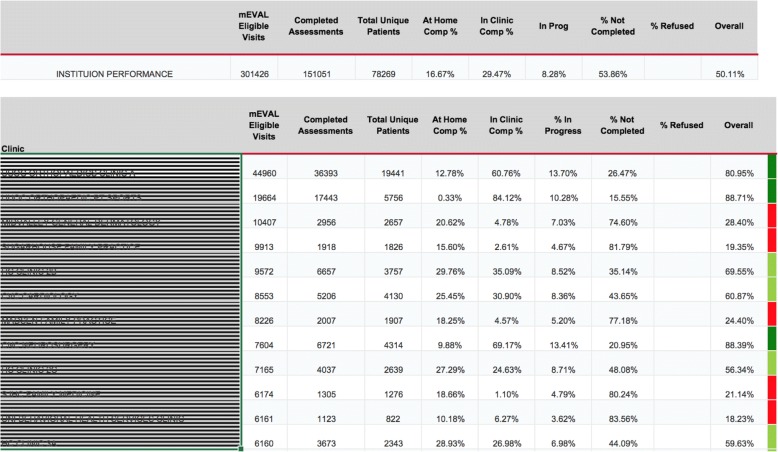
mEVAL Dashboard used to assess Completion Percentage across clinics and providers. Definitions: In Progress- patient did not completed all questions and no completion time was recorded. Completed- patient answered all question and completion time was recorded. Overall- summation of completed at home and in clinic, plus in progress or refused

Overall assessment completion percentage helped clinics track their progress (Fig. 3) and identify clinics with lower completion percentages that may need more support, training, or education. We provide PRO data to our clinicians for use in patient care and to researchers, with appropriate regulatory approval, for secondary use. Using these data, clinicians could also evaluate how different diseases impact quality of life domains. We created a self-service portal (Fig. 2) where providers and staff can view aggregated scoring information by clinic, assessment or instrument. This allows the user to compare different patient cohorts.

#### Organizational strategy

In order to ensure that all providers and staff understand the organizational focus of PROs, we worked with the marketing department to develop a strategy to inform clinicians and staff about PROs and how they fit into our institution. We have partnered with physician champions to tell their story of how PROs have changed the way they provide care for their patients to help promote adoption.

## Discussion

Our goal is to implement PROs in all ambulatory clinics at University of Utah Health, and to date we have achieved implementation in 75% of clinics, including Primary Care, Oncology, and Specialty clinics, such as Dermatology and Cardiology. Overall, our experiences can be summarized as followed:

- *Pre-implementation planning facilitates higher performance.* It was important for us to complete development, create a governance model, and coordinate a rollout process before we started using PROs. Creating these processes allowed for an expedited implementation schedule and prevented undue development troubleshooting.
- *A standardized process helps to streamline implementation.* A standardized process for implementation allowed for specific action items to be defined. For new issues identified during clinic implementation, solutions were added to the implementation checklist (Table 2), avoiding future problems.
- *Support from organizational leadership promotes adoption.* There needs to be support both from organizational and clinical leadership to allow for adoption at the provider level, culture change, and system-wide implementation, cultural changes.
- *Strong project collaboration is needed between several institutional groups.* In our healthcare system, we have several clinics with overlapping patient cohorts. These clinics have implemented similar assessments using identical instruments where applicable. This allows for ease of patient burden while maximizing use of data in our healthcare system.

In a 15 month period, we have implemented more than 205 unique instruments and collected more than 306,000 assessments from over 200,000 unique patients. We have demonstrated the ability for a large academic medical center to implement PROs and collect data on a large-scale basis. We plan to study the data collected within our health care system for clinical improvements and improve the value of care within our system. Our experience is similar to that of others that successfully implemented electronic PRO collection and provided real-time data to clinicians [20–23] and extends this work through closer clinical integration and broader implementation across diverse clinical and specialty settings.

While we are still working towards complete implementation and increased patient uptake, the principles identified and described provide a strong basis for continued refinement and success.

**Table 2:** Implementation Checklist

Implementation Check List	
Meet with clinic managers and providers to determine assessment build and intervals	
Set implementation date with programmers, clinic staff, send out calendar invites and email updates	
Order tablets, storing case	
Submit work order for smartphrase creation (shortcut tool used within our EHR to add scores to clinical note)	
Submit work order for assessment build	
Train providers and clinic staff	
Demo assessment with providers and make edits when necessary	
Ensure iPads, storing case, and training materials delivered to clinic	
Ensure staff has access to portal and dot phrase working properly	
Implementation, for all go-lives: be on site with breakfast, provide support and resources	

## Conclusion

It is feasible to implement PROs throughout a healthcare system, provided there is the full support of senior administration. The impact of our system-wide program for individual patient, point-of-care evaluation, as well as population health assessment will be assessed in future research. In order to determine greater feasibility it is necessary for other institutions to demonstrate the ability to implement a comprehensive program for the collection of PROs.

## Additional file

Additional file 1: Patient education website- https://healthcare.utah.edu/meval/. mEVAL Provider website: (DOCX 99 kb)

## Abbreviations

EHR: Electronic Health Record
FTE: Full-time equivalent
IT: Information Technology
mEVAL: My evaluation
PRO: Patient-reported outcomes
QR: Quick response
