# Supplementary material for: Patient reported outcomes – experiences with implementation in a University Health Care setting
Source: J Patient Rep Outcomes. 2018 Aug 17;2:34. doi: 10.1186/s41687-018-0059-0 (PMC6097980; doi:10.1186/s41687-018-0059-0)
Supplement: Supplementary file 1 [file 41687_2018_59_MOESM1_ESM.docx]

Supplementary Figure:

mEVAL Provider website
